# Multi-Mycotoxin Occurrence and Exposure Assessment Approach in Foodstuffs from Algeria

**DOI:** 10.3390/toxins12030194

**Published:** 2020-03-19

**Authors:** Choukri Khelifa Mahdjoubi, Natalia Arroyo-Manzanares, Nisserine Hamini-Kadar, Ana M. García-Campaña, Kihel Mebrouk, Laura Gámiz-Gracia

**Affiliations:** 1 Department of Analytical Chemistry, Faculty of Sciences, University of Granada, 18071 Granada, Spain; choukri_khelifa@hotmail.com (C.K.M.); amgarcia@ugr.es (A.M.G.-C.); 2 Department of Biology, Faculty of Natural and Life Science, University of Oran 1, 31100 Oran, Algeria; hamini.kadar@yahoo.fr (N.H.-K.); kihalm@gmail.com (K.M.); 3 Department of Analytical Chemistry, Faculty of Chemistry, University of Murcia, 30003 Murcia, Spain; natalia.arroyo@um.es

**Keywords:** Mycotoxins, cereals, UHPLC-MS/MS, exposure assessment, Algeria

## Abstract

A survey on 120 cereal samples (barley, maize, rice and wheat) from Algerian markets has been carried out to evaluate the presence of 15 mycotoxins (ochratoxin A, deoxynivalenol, fumonisin B1 and B2, T-2 and HT-2 toxins, zearalenone, fusarenon X, citrinin, sterigmatocystin, enniatins A, A1, B and B1, and beauvericin). With this purpose, a QuEChERS-based extraction and ultra-high performance liquid chromatography coupled to tandem mass spectrometry (UHPLC-MS/MS) were used. Analytical results showed that 78 cereal samples (65%) were contaminated with at least one toxin, while 50% were contaminated with three to nine mycotoxins. T-2 toxin, citrinin, beauvericin and deoxynivalenol were the most commonly found mycotoxins (frequency of 50%, 41.6%, 40.8% and 33.3%, respectively). Fumonisins (B1 + B2), enniatins B and B1, deoxynivalenol and zearalenone registered high concentrations (289–48878 µg/kg, 1.2–5288 µg/kg, 15–4569 µg/kg, 48–2055 µg/kg and 10.4–579 µg/kg, respectively). Furthermore, concentrations higher than those allowed by the European Union (EU) were observed in 21, 8 and 1 samples for fumonisins, zearalenone and deoxinivalenol, respectively. As a conclusion, the high levels of fumonisins (B1 + B2) in maize and deoxynivalenol, zearalenone and HT-2 + T-2 toxins in wheat, represent a health risk for the average adult consumer in Algeria. These results pointed out the necessity of a consistent control and the definition of maximum allowed levels for mycotoxins in Algerian foodstuffs.

## 1. Introduction

Mycotoxins are secondary metabolites of low molecular weight produced by a variety of fungi (mainly *Aspergillus, Fusarium* and *Penicillium* spp.). They present diverse chemical structures, having different biological effects on animals and humans, such as teratogenicity, carcinogenicity, mutagenicity, immunotoxicity or neurotoxicity [1,2]. These toxic compounds are often naturally occurring in the food chain and, therefore, result in human exposure either by direct consumption of contaminated crops, plants, seeds and fruits, or indirectly through ingestion of food derived from exposed animals (meat, eggs or milk) [3,4,5].

More than 400 mycotoxins have been reported so far, being aflatoxins (AFs), trichothecenes, fumonisins, zearalenone (ZEN) and ochratoxin A (OTA) the most representatives. However, other mycotoxins are becoming a global issue of high concern due to their extensive incidence in food and feed, including emerging *Fusarium* toxins, such as beauvericin (BEA) and enniatins (ENNs) [6,7,8].

Humans and animals are more frequently exposed to multiple mycotoxins than to a single one [9,10,11], producing an increasing concern about the health hazard. The combined effects of mycotoxins have been reported in several studies [12,13,14,15]. However, the actual health risk assessment is limited to their single effects, since there is not enough information available about the nature of the observed effects or the relative potencies of each mycotoxin in the mixture [16].

Due to the toxic effects of these compounds, the EU [17,18] and many countries have set maximum levels or recommendations in foodstuffs intended to human consumption (see Table 1). Moreover, for the most well-documented toxins, tolerable daily intakes (TDI) or provisional maximum tolerable daily intakes (PMTDI) have been established by the Food and Agriculture Organization (FAO)/World Health Organization (WHO) Joint Expert Committee on Food Additives (JECFA) [19,20].

Studies show that mycotoxins are ubiquitous contaminants [28,29]. It is estimated that they are present in approximately 25% of cereals consumed worldwide and a recent study suggests that this percentage could be even higher [30]. In general, mycotoxin contamination is higher when climatic conditions are favourable. Algeria is a North African country whose climate is characterised by high temperatures and high relative humidity in some areas that seems to stimulate the toxigenic moulds growth and toxinogenesis, in which cereals and cereal-based products represent a staple food for the population [31]. Moreover, no applicable norms concerning mycotoxin contamination of cereal (local or imported) have been set in Algeria. Indeed, a large amount of cereal commercialized in Algeria is imported and little is known about toxin contamination. Therefore, in order to the health risks associated with mycotoxin exposure different preventive approaches have been used. These include enforcement of legislation, good agricultural practices and the monitoring of mycotoxin contamination. But for this, it is essential to establish evidence and data on the presence of mycotoxins and exposure levels. Accurate exposure data on mycotoxins is an important input in risk assessment and management efforts as well as in the establishment of appropriated legislation for the monitoring and control of mycotoxin exposure in food [32].

In order to quantify the concentration of these hazards in different commodities, reliable and accurate analytical methods that allow their unambiguous identification and accurate quantification at low concentration are needed. In this sense, liquid chromatography (LC) or ultra-high performance LC (UHPLC) coupled to tandem mass spectrometry (MS/MS) have become the techniques of choice for the determination of multiple mycotoxins in food and feed [33,34,35]. In addition, alternative sample treatment methods, such as QuEChERS (acronym of Quick, Easy, Cheap, Effective, Rugged and Safe), are being increasingly applied to the analysis of mycotoxins, due to their feasibility, flexibility, versatility, low cost and rapidity [36].

Within this context, the main objective of this work is the validation of an analytical method based on a simple QuEChERS-based extraction and UHPLC–MS/MS for the determination of 15 mycotoxins, namely: OTA, deoxynivalenol (DON), fumonisin B1 (FB1) and B2 (FB2), T-2 and HT-2 toxins, ZEN, fusarenon X (F-X), citrinin (CIT), sterigmatocystin (STE), enniatin A (ENNA), A1 (ENNA1), B (ENNB) and B1 (ENNB1) and BEA. Secondly, the analysis of cereals samples (barley, maize, rice and wheat) collected from different Algerian markets will help to estimate the potential contribution to the dietary exposure of Algerian consumers.

## 2. Results

### 2.1. Method Validation 

The selected analytical method was validated for each cereal in terms of linearity, matrix effect, recovery, precision, limits of detection (LODs) and limits of quantification (LOQs). The results are summarised in the supplementary material (Table S1).

Method linearity was assessed by spiking blank samples at five concentration levels (processed in duplicate). All calibration curves showed a good linearity, with coefficients (R^2^) higher than 0.98 in all the cases. LODs and LOQs were determined as the concentration of analyte giving a signal to noise ratio (S/N) equal to 3 and 10, respectively. In all cases, LOQs were lower than maximum permitted or recommended concentrations established by the EU for those mycotoxins in cereals (FBs, DON, OTA, ZEN, T-2 and HT-2 toxin) [17,18].

Matrix effect was evaluated at the following concentration levels: OTA and STE: 25 μg/kg; CIT: 100 μg/kg; FB1, FB2, T-2, HT-2 and ZEN: 250 μg/kg; ENNB, ENNB1, ENNA, ENNA1 and BEA: 400 μg/kg; DON: 1000 μg/kg and F-X: 2500 μg/kg. Matrix effect was calculated as follow:

ME = 100 × (signal of spiked extract − signal of standard solution)/signal of standard solution

A strong ion suppression was observed for all the selected compounds in the four matrices under study, ranging from −77.8% to −18.2 % for barley, −76.5% to −15.6% for maize, −80.4% to −18.2% for rice and −76.9% to −14.3% for wheat (see supplementary material, Figure S1). As a consequence, calibration curves in matrix were used. 

The efficiency of the extraction process was evaluated by recovery studies, spiking blank samples at the same levels used in the matrix effect study. Each sample was processed in triplicate and injected three times. The ratio of peak areas of the samples spiked before extraction and the extracts spiked after extraction was used to calculate the recovery. The average recovery values were: 87.5% for barley, 84.8 % for maize, 88.5 % for rice and 86.2 % for wheat. 

Intra-day (repeatability) and inter-day precision (intermediate precision) were evaluated and expressed as relative standard deviation (%RSD). Spiked blank samples (processed and injected three times) at the same concentration levels mentioned above for the matrix effect and recovery studies were used. For the intra-day precision study, samples were analysed on the same day, while the inter-day precision was estimated through samples analysed on three consecutive days. The relative standard deviation (%RSD) for intra-day and inter-day precision were lower than 14% and 23%, respectively, for all the mycotoxins and matrix combination (see supplementary material Table S2). These values were all in a permitted range by European Commission [37].

### 2.2. Mycotoxins Occurrence Data

A total of 120 samples comprising barley (*n* = 30), maize (*n* = 30), rice (*n* = 30) and wheat (*n* = 30) were evaluated for the occurrence of mycotoxins (OTA, DON, FB1, FB2, T-2, HT-2, ZEN, F-X, CIT, STE, ENNA, ENNA1, ENNB, ENNB1 and BEA). Table 2 presents the occurrence, concentration range and mean concentration of each mycotoxin in positive cereal samples that is, considering only mycotoxins with a concentration above the LOQ.

Moreover, 78 out of 120 samples (65%) evidenced at least one mycotoxin above the LOQ, and 13 out of 15 mycotoxins included in the study were found in some of the analysed samples, being the exceptions OTA and STE (not detected). Overall, T-2, CIT, BEA and DON were the most commonly found mycotoxins with a global incidence of 50%, 40.8%, 38.3% and 33.3%, respectively. However, the maximum concentration value was found for FB1 in maize (42,143 µg/kg).

The results obtained for each mycotoxin (or group of mycotoxins) are commented below.

#### 2.2.1. Occurrence of Trichothecenes

Regarding the distribution of the studied trichothecenes (HT-2, T-2, DON and F-X), T-2 was the most frequently found, being present in 100% samples of maize and wheat at concentrations ranging from 16.6 to 47.2 µg/kg, being the mean concentrations (considering only positive samples) 24.9 µg/kg in maize and 21.8 µg/kg in wheat. HT-2 was present only in 7 wheat samples (23%) at concentrations from 8.4 to 36.7 µg/kg (mean value 18.1 µg/kg). None of the samples exceeded the maximum recommended concentration for these toxins in non-processed cereals (ranging from 100–1000 µg/kg for the sum of T-2 + HT-2) [17].

DON was found in 27 wheat samples (90%) and 13 maize samples (43.3%), with mean concentrations of positives samples of 588 µg/kg and 632 µg/kg, respectively. The highest concentration found for DON was 2055 µg/kg, corresponding to one maize sample that exceeded the maximum permitted concentration for DON established by the EU in maize (1750 µg/kg) [18]. As an example, a chromatogram of a wheat sample contaminated with DON is shown in supplementary material (Figure S2A).

F-X was determined in 3 samples of barley (10%), 24 of maize (80%) and 3 of wheat (10%). The maximum content of F-X was found in a maize sample (477 μg/kg).

In addition, the incidence of T-2 toxin in positive samples was higher than the incidence of DON in the same samples. Similar to our finding, Bouafifssa et al. [38] reported higher levels of T-2 and HT-2 compared to DON in Moroccan pasta, with contamination levels from 4 to 419 µg/kg and 4 to 50 µg/kg for T-2 and HT-2, respectively. The higher incidence of DON in maize and wheat compared to other cereals was also reported by Pleadin et al. [39] in 181 cereal samples from Croatia, where DON was found in 71%, 65%, 53% and 21% samples of maize, wheat, barley and oat, with mean concentrations of 1565, 223, 342 and 145 µg/kg, respectively. However, lower incidence and concentrations of DON, HT-2, T-2 and F-X were found in cereal samples from Italy, where mean levels in positive samples were 20.1, 4.8, 0.3 and 36.7 µg/kg, for DON, HT-2, T-2 and F-X respectively [40]. 

#### 2.2.2. Occurrence of Zearalenone

ZEN was found in 7 samples of maize (23.3%), 6 of rice (20%) and 19 of wheat (63.3%), being the mean concentrations of positive samples 109 µg/kg in maize, 9.9 µg/kg in rice and 102 µg/kg in wheat. One sample of maize (579 µg/kg) and 7 samples of wheat (with concentrations up to 295 µg/kg) exceeded the maximum permitted levels established in the EU for ZEN (350 µg/kg for maize and 100 µg/kg for other unprocessed cereals) [18].

The incidence of ZEN in our study was similar to those reported in other studies [41,42], but the contamination levels were lower than other values from literature. For instance, ZEN was found at concentrations up to 1399 µg/kg in cereals from Nigeria [43] and up to 15,700 µg/kg in maize samples from Belgium, where mean level of ZEN in the analysed samples was 2180 μg/kg [44]. However, other studies reported lower ZEN concentrations with an average of 12 and 14 µg/kg in Italian and Moroccan cereals, respectively [40,45].

#### 2.2.3. Occurrence of Fumonisins

FB1 and FB2 were found only in maize samples, with high concentrations and incidence rate. Thus, FB1 was present in 29 samples (96.6%) at concentrations from 289 up to 42,143 µg/kg, whereas FB2 was quantified in 27 samples (90%) at concentrations from 27.5 to 8603 µg/kg. The mean concentrations for positives samples were 14,812 and 2789 µg/kg for FB1 and FB2, respectively. Considering the sum of fumonisins (FB1 + FB2), it was in the range of 289–48,878 µg/kg. A total of 21 samples (70%) showed concentrations of (FB1 + FB2) above the maximum allowed level established by the EU (4000 µg/kg for unprocessed maize) [18] suggesting the high exposure of the population to these toxins.

According to the analyses, fumonisins were not detected in barley, rice and wheat samples. Such a trend was also observed by Ghali et al. in cereals from Tunisia, reporting the highest levels for fumonisins in maize samples at an incidence rate of 52% [46]. This confirms that the risk of fumonisin contamination of wheat, barley and rice is rather low due to the known tendency of the *Fusarium* spp. producing fumonisins (*F. verticillioides* and *F. proliferatum*) to infect maize [47]. This result is in agreement with other previous studies: thus, a high fumonisin incidence was reported in Nigerian maize-based products with concentrations ranging from 74 to 22064 µg/kg [43], in maize from South Africa, reporting concentrations up to 53863 µg/kg for FB1 [48], and from Ethiopia, where 77% samples of maize contaminated with fumonisins at concentrations between 25–4500 µg/kg, were attributed to *F. verticillioides* [49].

#### 2.2.4. Occurrence of Citrinin

Remarkably, 40.8% of the total analysed samples were contaminated by CIT; it was present in 9 (30%), 25 (83.3%) and 15 (50%) samples of barley, maize and wheat, respectively, showing mean levels of 26.2 µg/kg in barley, 32.7 µg/kg in maize and 16.8 µg/kg in wheat samples. The highest concentration of CIT was found in a maize sample (273 µg/kg). CIT was not detected in rice samples. 

These results may be explained by the susceptibility of the analysed cereals (barley, maize and wheat) to CIT-producing fungi (*Aspergillus* and *Penicillium* spp.) and to the influence of climatic conditions such as substrate composition, temperature and water activity (aw) to enhance CIT production, especially during storage, as it is well-known that mycotoxin production is modulated by environmental factors [50].

#### 2.2.5. Occurrence of Emerging Mycotoxins

Only maize and wheat samples were contaminated with BEA and ENNs, while these emerging mycotoxins were not detected in rice and barley. ENNB1 was the most frequent ENN (2 samples (6.6%) of maize and in 21 samples (70%) of wheat) with concentrations from 15.0–107 µg/kg and 19.5–4569 µg/kg for maize and wheat, respectively. ENNA1 was quantified in 3 samples (10%) of maize and 14 samples (46.7) of wheat, with mean concentrations for positive samples of 56.4 µg/kg and 107 µg/kg, respectively. ENNA and ENNB were found in 7 (23.3%) and 18 samples (60%) of wheat, respectively. The contamination levels varied between 8.4 and 87.6 µg/kg for ENNA and from 1.2 to 5288 µg/kg for ENNB, with mean values for positive samples of 28.3 and 1668 µg/kg, respectively. ENNA and ENNB were not detected in maize. A chromatogram of a wheat sample contaminated with ENNB1 is shown in supplementary material (Figure S2B).

Concerning BEA, 25 samples (83.3%) of maize and 21 samples (70%) of wheat were positives, with concentrations between 0.85–31.4 µg/kg and 2.8–486 µg/kg in maize and wheat samples, respectively.

Other previous works including determination of ENNs and BEA in cereals also showed the high incidence of these mycotoxins. Thus, a high incidence of BEA (80%) was recently reported in Serbian maize with levels ranging from 8 to 129 µg/kg [7]. Moreover, a study on the occurrence of emerging mycotoxins in Spanish cereals showed high incidence of ENNs (73.4%), wherein ENNA1 was the most frequent emerging mycotoxins, with the highest concentrations (33.3–814 mg/kg) [51]. Furthermore, the high contamination of BEA and ENNs was also reported in cereals from Italy [40] and up to 800 mg/kg of ENNB1 were found in a wheat-based cereal sample from Morocco [8]. In contrast with our results, Oueslati et al. [52] did not detect BEA in maize and wheat from Tunisia.

#### 2.2.6. Co-Occurrence of Mycotoxins in Analysed Samples

The co-occurrence of mycotoxins as well as the main combinations found in the analysed cereal samples were evaluated (see supplementary material Table S3). Among the positive samples, 50% (all maize (*n* = 30) and wheat (*n* = 30) samples) were found to be contaminated with more than one mycotoxin. The most frequent co-occurrence was the combination of 5 mycotoxins for maize and 8 mycotoxins for wheat. Moreover, different combinations were observed, depending on the cereal, the most frequent combinations being: (FB1 + FB2 + T-2 + F-X + CIT + BEA) and (FB1 + FB2 + T-2 + F-X + BEA) in maize, and (DON + T-2 + ZEN + ENNA1 + ENNB + ENNB1) in wheat samples. The highest number of mycotoxins occurring simultaneously was nine in 2 maize samples (DON + FB1 + FB2 + T-2 + ZEN + F-X + CIT + BEA + ENNA1) and 1 wheat sample (DON + HT-2 + T-2 + ZEN + CIT + BEA + ENNA1 + ENNB + ENNB1). 

The co-occurrence of mycotoxins in cereals has been studied previously, especially in the Mediterranean area [38,40,52]. In agreement with our results, in wheat grains from Morocco, 51% samples were contaminated with 2–6 mycotoxins [53], whereas that at least one mycotoxin was present in the 65% cereal-derived samples from Spain [54]. Recently, a study performed in Italy showed that 81% cereal samples were contaminated with more than one mycotoxin and the most frequent co-occurrence was with DON, F-X, ENNB and ENNA1 [40]. A summary of the results obtained in this study and other previously reported occurrence studies are presented in Table 3. 

These results demonstrated that it is not unusual to find cereals contaminated with several mycotoxins, and evidenced the human exposure to multiple mycotoxins [9,11,12,13]. Therefore, these findings point out the necessity of more toxicity studies that consider co-exposure to multiple mycotoxins, to detect possible synergism and additive effects and its consequent potential impact for public health.

### 2.3. Exposure Estimates

The dietary exposure to the studied mycotoxins was evaluated by calculating the probable daily intake (PDI), which combines mycotoxins analysis data obtained from the analysed samples with the food consumption of the adult population with a body weight of 60 kg [55].

The PDI (µg/kg per body weight (bw)/day) of each mycotoxin was calculated using the following equation [55]:

PDI = (Cm × K)/bw(1)

where C_m_ is the mean content of a mycotoxin in the cereal (µg/kg); K is the average consumption of the commodity (g/day) and bw is the body weight used for adult population.

Once the PDI had been calculated, the health risk characterization of each mycotoxin (% of relevant TDI) was estimated as the ratio of PDI to TDI (µg/kg bw/day) for each mycotoxin as follows:

%TDI = (PDI/TDI) × 100(2)

The PMTDI or TDI and the provisional tolerable weekly intake (PTWI) set by both the FAO/WHO JECFA and the Scientific Committee on Food (SCF), were used as reference doses [19,20]. Data on consumption of barley (36 g/day), maize (44 g/day), rice (8 g/day) and wheat (502 g/day) by Algerian population were mainly obtained from FAO statistical study [56]. The results obtained are summarised in Table 4.

From the PDI values, it can be concluded that maize and wheat samples represent an important dietary exposure of mycotoxins for the Algerian population, with a dietary exposure range of 2.8 × 10^−3^–12.91 (µg/kg bw/day) and 0.14–13.96 (µg/kg bw/day) in maize and wheat, respectively. In these samples, the obtained values for FB1 (10.86 µg/kg bw/day) in maize and ENNB (13.96 µg/kg bw/day) in wheat were the highest contribution for the PDI of mycotoxins for the Algerian population.

The exposure assessment was evaluated for all the mycotoxins with TDI available, namely DON, FB1+ FB2, ZEN, T-2 + HT-2 (see Table 4). The results showed that Algerian consumers present at a high risk of exposure to the sum of fumonisins (FB1 + FB2) through maize consumption (%TDI of 645.4), and to DON, ZEN and the sum of (HT-2 + T-2) through wheat consumption with %TDI of 491.9, 341.4 and 333.8, respectively. These values are several-hundred fold higher than the values established by JECFA for FB1 + FB2 (PMTDI of 2 µg/kg bw/day), DON (PMTDI of 1 µg/kg bw/day), ZEN (TDI of 0.25 µg/kg bw/day) and for the sum of HT-2 and T-2 toxins (TDI of 0.1 µg/kg bw/day).

These findings suggest that the intake of mycotoxins from analysed maize and wheat samples represent a high health risk for the average adult consumers in Algeria and pointed towards the necessity for a consistent control over these contaminants.

These results are globally in line with other studies from African countries; for instance, a study performed in Tanzania reported that fumonisin exposures to adult individuals in 38% of the households exceeded the provisional maximum tolerable daily intake (PMTDI) of 2 µg/kg bw based on the fumonisins (FB1 + FB2) concentration of up to 11 mg/kg in the maize grains [57]. Similar results were found for fumonisins (FB1 + FB2 + FB3) dietary exposure of 12.4 µg/kg bw/day in Nigerian infants and 8.2 µg/kg bw/day in children by maize intake with %TDI of 622.2 and 414.8, which were 311 and 207 times higher than the tolerable daily intake of 2 µg/kg bw/day [58].

Moreover, high attention should be devoted to the health risk scenarios for consumers in the case of co-exposures to multiple mycotoxins. However, the effect of co-occurrence of mycotoxins have not been well understood yet. These effects could be additive, synergistic and can vary with dose, exposure time, and toxicological end point [9].

## 3. Conclusions

In this study, a LC-MS/MS analytical method for determination of 15 mycotoxins in cereals has been applied to provide data on the occurrence of these hazards in barley, maize, rice and wheat from Algeria. Moreover, the risk associated with the exposure to mycotoxins through intake of cereal products has been estimated.

Results on mycotoxins occurrence showed that 65% of the samples were contaminated with at least one mycotoxin. The maximum acceptable levels established in EU where exceeded in 21 samples (70%) for fumonisins (4000 µg/kg of FB1 + FB2 in maize), 8 samples for ZEN (100 µg/kg for maize and 350 for other unprocessed cereals) and 1 sample for DON (1750 µg/kg for wheat and maize) [18]. Co-contamination was observed in 50% of the analysed samples (all maize and wheat samples). Among them, some samples were contaminated with up to 9 mycotoxins. Due to these high levels of contamination, this study concluded that the Algerians are at high risk of exposure to the sum of fumonisins (FB1 + FB2) through the consumption of maize, and to DON, ZEN and the sum of (HT-2 +T-2) through the consumption of wheat. Therefore, preventive approaches to curtail health risks associated with mycotoxins exposures are needed, which requires, at first instance, government intervention. The analytical results of this survey should encourage Algerian authorities to introduce allowed maximum limits of mycotoxins in cereals. In our opinion, continuous monitoring of these mycotoxins with a higher number of samples and other susceptible foodstuffs (such as cereal-based products, nuts or dried fruits) are recommended to assess the situation, at least for such a time until proper regulatory limits are set. On the other hand, the focus should also be directed towards reduction and control of mycotoxins producing fungi in the food chain.

## 4. Materials and Methods 

### 4.1. Reagents and Materials

Methanol (MeOH) and acetonitrile (MeCN) of LC-MS grade, and ammonium formate were supplied by VWR International Eurolab S.L. (Barcelona, Spain). Formic acid eluent additive for LC–MS was obtained from Sigma Aldrich (St. Louis, MO, USA). Magnesium sulphate (MgSO_4_), sodium chloride (NaCl) and sodium citrate were purchased from Panreac Química (Barcelona, Spain), while disodium hydrogen citrate sesquihydrate was supplied by Merck (Darmstadt, Germany). Ultrapure water was obtained from a Milli-Q Plus system (Millipore Bedford, MA, USA).

Mycotoxin standard solutions (10 mg/L in MeCN) of OTA, STE, F-X, DON, CIT, ZEN, FB1, FB2, T-2 and HT-2 were purchased from Techno Spec (Barcelona, Spain). Individual standards (powder) of ENNA, ENNA1, ENNB, ENNB1 and BEA were obtained from Sigma Aldrich and stock solutions were prepared at 1000 mg/L in MeCN. Multi-mycotoxins intermediate working solutions in MeCN (1 mg/L of OTA and STE; 2 mg/L of CIT; 10 mg/L of FB1, FB2, T-2, HT-2 and ZEN; 100 mg/L of DON, ENNA, ENNA1, ENNB, ENNB1 and BEA and 1000 mg/L of F-X) were prepared by combining suitable aliquots of each individual standard stock solution. These solutions were stored at −20 °C.

Nylon syringe filters (13 mm, 0.22 μm, from VWR) were used for filtration of extracts prior to the injection into the chromatographic system.

### 4.2. Instruments and Equipment

UHPLC-MS/MS analyses were performed in an Agilent 1290 Infinity LC (Agilent Technologies, Waldbronn, Germany) coupled to an API 3200 triple quadrupole mass spectrometer (AB Sciex, Darmstadt, Germany) with electrospray ionization (ESI). The chromatographic separation was performed using an Agilent Zorbax Eclipse Plus RRHD C18 column (50 × 2.1 mm, 1.8 µm). Analyst software (Version 1.6.3, AB Sciex, Darmstadt, Germany) was used for acquisition and data analysis.

During the sample treatment, an evaporator System (System EVA-EC, from VLM GmbH, Bielefeld, Germany), a vortex-2 Genie (Scientific Industries, Bohemia, NY, USA), a universal 320R centrifuge (Hettich ZENtrifugen, Tuttlingen, Germany), and a kitchen blender were used.

### 4.3. Samples

A total of 120 cereal samples (barley, maize, rice and wheat) destined for human consumption were randomly purchased from different local markets in three areas of the western region of Algeria—Aint Temouchent, Oran and Tiaret—during the year 2018 (see Table 5). In order to obtain representative samples, several sub-samples were taken from each batch, being thoroughly mixed to achieve a final 1-kg sample. Finally, the samples were grinded, homogenized and stored in a dark and dry place until analysis.

### 4.4. Mycotoxins Extraction Procedure 

**Figure 1:**
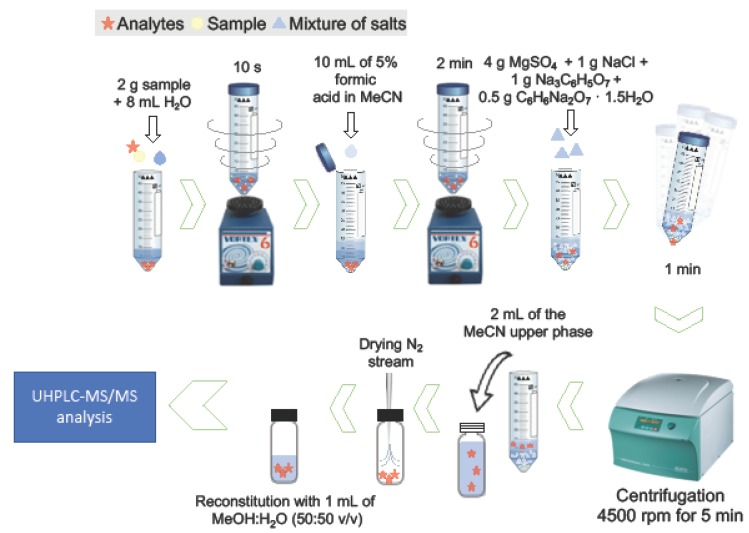
Graphical scheme of the extraction procedure.

A previous method was used for the extraction of mycotoxins in the different samples [59]. Briefly, 2 g of grounded sample were weighed in a polypropylene centrifuge tube (50 mL), 8 mL of water was added, and the mixture was vortexed for 10 s. Subsequently, 10 mL of 5% formic acid in MeCN was added to the tube, shaking by vortex for 2 min. Then, 4 g of MgSO_4_, 1 g of NaCl, 1 g of sodium citrate and 0.5 g of disodium hydrogen citrate sesquihydrate were added and the tube was shaken vigorously for 1 min. After centrifugation at 4500 rpm (3722× *g*) for 5 min, 2 mL of the upper supernatant layer was transferred to a 4-mL vial, evaporated to dryness under a gentle stream of nitrogen, and reconstituted to a final volume of 1 mL with a mixture of MeOH:water (50:50, *v*/*v*). The samples were filtered before injection and the 15 mycotoxins were determined by UHPLC-MS/MS. A graphical scheme of the procedure is shown in Figure 1.

### 4.5. Ultra-High Performance Liquid Chromatography Coupled to Tandem Mass Spectrometry (UHPLC-MS/MS) Analysis 

Chromatographic analyses were performed using a gradient elution with water (phase A), and MeOH (phase B), both containing 0.3% formic acid and 5 mM ammonium formate, at a flow rate of 0.4 mL/min. The gradient elution program was as follows: 0–1 min, 5% B; 4 min, 50% B; 5 min, 80% B; 5.5 min, 90% B; 5.7 min, 5% B; 5.7–8 min, 5% B. The temperature of the column was kept at 35 °C and the injection volume was 5 µL.

The electrospray ionization was carried out in in the positive mode and the acquisition was performed under multiple reaction monitoring (MRM) conditions. The ionization source parameters were set as follows: source temperature: 500 °C; curtain gas (nitrogen): 30 psi; ion spray voltage: 5000 V; and GAS 1 and GAS 2 (both of them nitrogen): 50 psi. The applied cone voltages and collision energies for each mycotoxin are summarized in the supplementary material (Table S4). In all cases, the most abundant product ion was used for quantification, while the second one was used for confirmation. 

## Figures and Tables

**Table 1:** Maximum tolerable levels set for some mycotoxins in cereals.

Mycotoxin	Commodity	Maximum Level (µg/kg)
	**European Union [17,18]**	
Aflatoxin B1	Cereals and cereal products	2
Aflatoxins ^a^	Cereals and cereal products	4
	Maize	10
Ochratoxin A	Unprocessed cereals	5
Zearalenone	Unprocessed maize	350
	Unprocessed cereals other than maize	100
Fumonisins B1 + B2	Unprocessed maize	4000
Deoxynivalenol	Unprocessed cereals other than durum wheat, oats, maize	1250
	Unprocessed durum wheat, oats and maize	1750
T-2 + HT-2	Unprocessed barley and maize	200
	Unprocessed oats	1000
	Unprocessed wheat, rye and other cereals	100
	**United States of Americ [21,22]**	
Aflatoxin B1	All food crops	15
Fumonisins B1 + B2 + B3	Unprocessed maize	4000
	**Canada [23]**	
Deoxynivalenol	Unprocessed wheat	2000
	**Japan [24,25]**	
Aflatoxin B1	All food crops	10
Deoxynivalenol	Unprocessed wheat	1100 ^b^
	**China [26]**	
Aflatoxin B1	Unprocessed maize	20
	Rice (brown rice)	10
	Unprocessed wheat barley, other cereals	5
Deoxynivalenol	Maize, barley, wheat, other cereals	1000
Ochratoxin A	Unprocessed cereals	5
Zearalenone	Unprocessed wheat and maize	60
	**Morocco [27]**	
Aflatoxin B1	Cereals and cereal products	2
Aflatoxins ^a^	Cereals and cereal products	4
Ochratoxin A	Unprocessed cereals	5
Deoxynivalenol	Unprocessed durum wheat, oats and maize	1750
Zearalenone	Unprocessed maize	200

^a^ AFs: Sum of AFB1 + AFB2 + AFG1 + AFG2; ^b^ Provisional maximum level.

**Table 2:** Summary of mycotoxins found in the analysed cereal samples.

	Barley (*n* = 30)				Maize (*n* = 30)				Rice (*n* = 30)				Wheat (*n* = 30)			
	I (%) ^a^	Mean ^b^	LOD-LOQ ^c^	Range ^d^	I (%) ^a^	Mean ^b^	LOD-LOQ ^c^	Range ^d^	I (%) ^a^	Mean ^b^	LOD-LOQ ^c^	Range ^d^	I (%) ^a^	Mean ^b^	LOD-LOQ ^c^	Range ^d^
Analytes		(µg/kg)				(µg/kg)				(µg/kg)				(µg/kg)		
**FB1**	nd	nd	–	–	29 (96.6)	14,812		289–42,143	nd	nd	–	–	nd	nd	–	–
**FB2**	nd	nd	–	–	27 (90)	2789	–	27.5–8603	nd	nd	–	–	nd	nd	–	–
**HT-2**	nd	nd	9	–	nd	nd	–	–	nd	nd	–	–	7 (23)	18.1	3	8.4–36.7
**T-2**	nd	nd	–	–	30 (100)	24.9	–	24.6–25.7	nd	nd	–	–	30 (100)	21.8	–	16.6–47.2
**DON**	nd	nd	–	–	13 (43)	632	–	47.6–2055	nd	nd	–	–	27 (90)	588	1	68.3–1363
**ZEN**	nd	nd	2	–	7 (23.3)	109	–	20.4–579	6 (20)	9.9	17	8.6–15.5	19 (63.3)	102	9	9.6–295
**F-X**	3 (10)	190	2	142–284	24 (80)	281	1	177–477	nd	nd	–	–	3 (10)	152	7	139–159
**OTA**	nd	nd	–	–	nd	nd	–	–	nd	nd	–	–	nd	nd	–	–
**CIT**	9 (30)	26.2	2	10.9–52.0	25 (83.3)	32.7	–	8.6–273	nd	nd	23	–	15 (50)	16.8	14	9.8–32.3
**STE**	nd	nd	–	–	nd	nd	–	–	nd	nd	–	–	nd	nd	–	–
**BEA**	nd	nd	–	–	25 (83.3)	3.8	4	0.85–31.4	nd	nd	–	–	21 (70)	155.4	–	2.8–486
**ENNA**	nd	nd	–	–	nd	nd	–	–	nd	nd	–	–	7 (23.3)	28.3	–	8.4–87.6
**ENNA1**	nd	nd	–	–	3 (10)	56.4	–	11.5–103	nd	nd	–	–	14 (46.7)	107	3	4.0–395
**ENNB**	nd	nd	–	–	nd	nd	–	–	nd	nd	–	–	18 (60)	1668	–	1.2–5288
**ENNB1**	nd	nd	–	–	2 (6)	60.9	–	15.0–107	nd	nd	–	–	21 (70)	469	–	19.5–4569
**Total**	**12 (40)**				**30 (100)**				**6 (20)**				**30 (100)**			

^a^ Incidence of samples ≥ LOQ (% of samples ≥ LOQ), ^b^ Mean value for samples ≥ LOQ, ^c^ Number of samples ≥ LOD and ≤ LOQ, ^d^ minimum value—maximum value, *n*: Number of samples; nd: Not detected.

**Table 3:** Occurrence of mycotoxins in cereals from different surveys.

	**Barley (*n* = 30)**			**Maize (*n* = 30)**			**Rice (*n* = 30)**			**Wheat (*n* = 30)**			**Analytical Method**	**LOQ (µg/kg)**	**Ref.**
	**I (%) ^a^**	**Range ^c^ (μg/kg)**	**Mean ^b^ (μg/kg)**	**I (%) ^a^**	**Range ^c^ (μg/kg)**	**Mean ^b^ (μg/kg)**	**I (%) ^a^**	**Range ^c^ (μg/kg)**	**Mean (μg/kg)**	**I (%) ^a^**	**Range ^c^ (μg/kg)**	**Mean ^b^ (μg/kg)**			
**FB1**	nd	–	nd	29 (96.6)	289–42,143	14812	nd	–	nd	nd	–	nd	Ultra-high performance liquid chromatography coupled to tandem mass spectrometry (UHPLC-MS/MS)	2.6–4.8	This work
**FB2**	nd	–	nd	27 (90)	27.5–8603	2789	nd	–	nd	nd	–	nd		2.2–10	
**HT–2**	nd	–	nd	nd	–	nd	nd	–	nd	7 (23)	8.4–36.7	18.1		2.8–9.9	
**T–2**	nd	–	nd	30 (100)	24.6–25.7	24.9	nd	–	nd	30 (100)	16.6–47.2	21.8		2.3–4.4	
**DON**	nd	–	nd	13 (43)	47.6–2055	632	nd	–	nd	27 (90)	68.3–1363	588		4.2–4.8	
**ZEN**	nd	–	nd	7 (23.3)	20.4–579	109	6 (20)	8.6–15.5	9.9	19 (63.3)	9.6–295	102		4.3–9.7	
**F–X**	3 (10)	142–284	190	24 (80)	177–477	281	nd	–	nd	3 (10)	139–159	152		90–174	
**OTA**	nd	–	nd	nd	–	nd	nd	–	nd	nd	–	nd		20–92	
**CIT**	9 (30)	10.9–52.0	26.2	25 (83.3)	8.6–273	32.7	nd	–	nd	15 (50)	9.8–32.3	16.8		8.4–23	
**STE**	nd	–	nd	nd	–	nd	nd	–	nd	nd	–	nd		0.6–1.3	
**BEA**	nd	–	nd	25 (83.3)	0.85–31.4	3.8	nd	–	nd	21 (70)	2.8–486	155.4		0.6–1.3	
**ENNA**	nd	–	nd	nd	–	nd	nd	–	nd	7 (23.3)	8.4–87.6	28.3		0.5–1.2	
**ENNA1**	nd	–	nd	3 (10)	11.5–103	56.4	nd	–	nd	14 (46.7)	4.0–395	107		1.4–2.7	
**ENNB**	nd	–	nd	nd	–	nd	nd	–	nd	18 (60)	1.2–5288	1668		1.2–3.8	
**ENNB1**	nd	–	nd	2 (6)	15.0–107	60.9	nd	–	nd	21 (70)	19.5–4569	469		2.6–4.4	
	**Barley (*n* = 9)**			**Oat (*n* = 7)**			**Rye (*n* = 11)**			**Wheat (*n* = 57)**			**Analytical Method**	**LOQ (µg/kg)**	**Ref.**
	**I (%) ^a^**	**Range ^c^ (μg/kg)**	**Mean ^b^ (μg/kg)**	**I (%) ^a^**	**Range ^c^ (μg/kg)**	**Mean ^b^ (μg/kg)**	**I (%) ^a^**	**Range ^c^ (μg/kg)**	**Mean (μg/kg)**	**I (%) ^a^**	**Range ^c^ (μg/kg)**	**Mean ^b^ (μg/kg)**			
**DON**	1 (11)	up to 35.5		4 (57)	10.3–83	29.9	5 (45.5)	16.5–79.6	23.23	16 (28)	9.6–99.6	10.96	LC– MS/MS	10	[40]
**3–AcDON**	nd	–	nd	1 (14.2)	5.23	5.24	nd	–	nd	nd	–	nd		15	
**15–AcDON**	nd	–	nd	nd	–	nd	nd	–	nd	2 (3.5)	10.8–29.13	0.64		15	
**FUS–X**	4 (44)	27.5–47.3	18.43	3 (42.8)	26–75	23	5 (45.5)	42.4–70.2	28.52	14 (24)	12.50–102	18.44		15	
**NIV**	3 (33)	21.7–106	25.15	4 (57)	45.5–50.4	27.13	2 (18)	33.9–34.4	56.9	11 (19)	12–106	8.86		15	
**DAS**	nd	–	nd	nd	–	nd	nd	–	nd	nd	–	nd		10	
**NEO**	nd	–	nd	nd	–	nd	nd	–	nd	nd	–	nd		5	
**HT–2**	nd	–	nd	nd	–	nd	3 (27.2)	6.98–50.3	5.34	3 (5.2)	6.78–60.10	4.44		10	
**T–2**	nd	–	nd	nd	–	nd	nd	–	nd	2 (3.5)	7.14–17.8	0.39		5	
**ZEN**	2 (22)	11.15	11.16	nd	–	nd	nd	–	nd	5 (8.7)	2.35–27.15	12.17		5	
**α –ZEN**	nd	–	nd	nd	–	nd	nd	–	nd	nd	–	nd		5	
**β –ZEN**	nd	–	nd	nd	–	nd	nd	–	nd	nd	–	nd		5	
**BEA**	nd	–	nd	4 (57)	7.2–41	8.8	5 (45.5)	8.9–16.5	2.72	5 (8.7)	9.6–35	12.8		15	
**ENNB**	nd	–	nd	3 (42.8)	5.5–97	2.8	6 (54.4)	6.7–45	5.8	16 (28)	5.5–97	20.2		15	
**ENNB1**	2 (22)	5.5–7.3	1.4	nd	–	nd	nd	–	nd	2 (3.5)	5.47–33.1	0.43		15	
**ENNB4**	8 (88.9)	6.6–60	15.7	4 (57)	20–284.2	50.7	4 (36.3)	23.4–74	13.8	18 (31)	5.7–110.2	38.44		15	
**ENNA**	nd	–	nd	nd	–	nd	4 (36.3)	7.8–9.8	7.1	6 (10)	8.4–29.8	1.56		10	
**ENNA1**	nd	–	nd	2 (28.5)	9–45.5	8.7	nd	–	nd	11 (19)	5.3–55	0.74		15	
	**Barley (*n* = 5)**			**Sorghum (*n* = 3)**			**Processed cereals (*n* = 13)**			**Wheat (*n* = 34)**			**Analytical Method**	**LOQ (µg/kg)**	**Ref.**
	**I (%) ^a^**	**Range ^c^ (μg/kg)**	**Mean ^b^ (μg/kg)**	**I (%) ^a^**	**Range ^c^ (μg/kg)**	**Mean ^b^ (μg/kg)**	**I (%) ^a^**	**Range ^c^ (μg/kg)**	**Mean (μg/kg)**	**I (%) ^a^**	**Range ^c^ (μg/kg)**	**Mean ^b^ (μg/kg)**			
**AFB1**	nd	–	nd	66.6	14.4–79.9	46.7	nd	–	nd	nd	–	nd	UHPLC–MS/MS	1	[52]
**AFG2**	4 (80)	23.1–52.4	35.5	3 (100)	13–36.8	24.6	nd	–	nd	4 (11)	5.2–8.7	6.6		1	
**HT–2**	3 (60)	4.9–11	7.6	nd	–	nd	nd	–	nd	4 (11)	5.3–7.1	5.8		5	
**FB1**	1 (20)	up to 63.1	na	2 (66.6)	6.4–120	na	nd	–	nd	nd	–	nd		5	
**FB2**	nd	–	nd	1 (33.3)	61.5	61.5	1 (7.6)	6.4	6.4	1 (3)	8.7	8.7		5	
**OTA**	nd	–	nd	nd	–	nd	1 (7.6)	5	5	nd	–	nd		5	
	**Barley (*n* = 4)**			**Maize (*n* = 28)**			**Rice (*n* = 1)**			**Wheat (*n* = 21)**			**Analytical Method**	**LOQ (µg/kg)**	**Ref.**
	**I (%) ^a^**	**Range ^c^ (μg/kg)**	**Mean ^b^ (μg/kg)**	**I (%) ^a^**	**Range ^c^ (μg/kg)**	**Mean ^b^ (μg/kg)**	**I (%) ^a^**	**Range ^c^ (μg/kg)**	**Mean (μg/kg)**	**I (%) ^a^**	**Range ^c^ (μg/kg)**	**Mean ^b^ (μg/kg)**			
**ENNA**	nd	–	nd	nd	–	nd	I (%)^a^	Range^b^ (μg/kg)	Mean^c^ (μg/kg)	nd	–	nd	LC–DAD	6	[51]
**ENNA1**	na	up to 361,570	148,160	na	up to 813,010	167,700	nd	–	nd	na	up to 634,850	225370		4	
**ENNB**	1 (25)	up to 21,370	21370	na	up to 6310	4470	1(100)	up to 814,410	814420	nd	–	nd		4	
**ENNB1**	1 (25)	up to 4340	4340	na	up to 21,370	21370	1(100)	up to 7950	7950	nd	–	nd		5	
**BEA**	2 (50)	up to 6940	4870	6 (21.4)	up to 9310	5720	nd	–	nd	9 (42)	up to 3500	2300		5	
**FUS**	nd	–	nd	na	up to 2470	2470	1(100)	up to 11780	11780	na	up to 6630	3120		6	
	**Maize (*n* =136)**			**Sorghum (*n* =110)**			**Millet (*n* = 87)**			**Ogi (*n* = 30)**			**Analytical Method**	**LOQ (µg/kg)**	**Ref.**
	**I (%) ^a^**	**Range ^c^ (μg/kg)**	**Mean ^b^ (μg/kg)**	**I (%) ^a^**	**Range ^c^ (μg/kg)**	**Mean ^b^ (μg/kg)**	**I (%) ^a^**	**Range ^c^ (μg/kg)**	**Mean (μg/kg)**	**I (%) ^a^**	**Range ^c^ (μg/kg)**	**Mean ^b^ (μg/kg)**			
**FB1**	(65)	up to 8222	541	(8)	up to 78	64	(9)	up to 18.172	2333	(93)	up to 1903	590	LC–MS/MS	16.4–20	[43]
**FB2**	(54)	up to 2885	376	(2)	up to 55	48	(13)	up to 3892	609	(87)	up to 1283	472		22.6–24.2	
**FB3**	(43)	up to 445	117	(2)	up to 46	38	nd	–	nd	(77)	up to 371	121		28	
**DON**	(16)	up to 225	99	(3)	up to 119	100	(13)	up to 543	151	(13)	up to 74	61		14–24	
**15–AcDON**	nd	–	nd	(2)	up to 44	39	(1)	up to 11	11	nd	–	nd		8.8–14	
**DON–3G**	nd	–	nd	(23)	up to 63	24	nd	–	nd	(17)	up to 44	30		7.54–30.6	
**ZEN**	(1)	up to 65	65	(1)	up to 38	38	(14)	up to 1399	419	(3)	up to 39	39		6.5–7.7	
**ZEN–14G**	(9)	up to 24	21	(3)	up to 22	19	(6)	up to 34	23	(3)	up to 31	31		9.2–10.2	
**α –ZEN**	(1)	up to 20	20	(3)	up to 33	33	nd	–	nd	(7)	up to 22	20		10–14	
**β** **–ZEN**	(2)	up to 21	20	(1)	up to 21	21	(1)	up to 39	39	(10)	up to 20	19		14.4–16	
**HT–2**	(1)	up to 20	20	(8)	up to 31	20	(5)	up to 36	36	(3)	up to 13	13		13	
**NIV**	(2)	up to 271	206	nd	–	nd	nd	–	nd	(7)	up to 160	148		175–162.6	
**FUS–X**	(1)	up to 154	154	nd	–	nd	nd	–	nd	(7)	up to 133	137		41.2–147.2	
**DAS**	(13)	up to 8	3	(18)	up to 16	5	(29)	up to 25	5	nd	–	nd		0.64–1	
	**Barley (*n* = 34)**			**Maize (*n* = 63)**			**Oats (*n* = 33)**			**Wheat (*n* = 51)**			**Analytical Method**	**LOQ (µg/kg)**	**Ref.**
	**I (%) ^a^**	**Range ^c^ (μg/kg)**	**Mean ^b^ (μg/kg)**	**I (%) ^a^**	**Range ^c^ (μg/kg)**	**Mean ^b^ (μg/kg)**	**I (%) ^a^**	**Range ^c^ (μg/kg)**	**Mean (μg/kg)**	**I (%) ^a^**	**Range ^c^ (μg/kg)**	**Mean ^b^ (μg/kg)**			
**DON**	(53)	74–228	342	(71)	215–2942	1565	(21)	34–201	145	(65)	115–278	223	ELISA–UV	20.5	[39]
**ZEN**	(9)	5 –68	32	(78)	10–611	187	(6)	4–43	17	(69)	7–107	56		2.1	
**FUS–X**	(15)	25–121	44	(90)	37–4434	1756	(6)	25–31	28	(39)	28–203	66		24.5	
**T–2**	(32)	5– 26	13	(57)	5–42	24	(18)	5–10	7	(25)	6–18	9		4.1	
	**Barley (*n* = 20)**			**Maize (*n* = 20)**			**Wheat (*n* =20)**						**Analytical Method**	**LOQ (µg/kg)**	**Ref.**
	**I (%) ^a^**	**Range ^c^ (μg/kg)**	**Mean ^b^ (μg/kg)**	**I (%) ^a^**	**Range ^c^ (μg/kg)**	**Mean ^b^ (μg/kg)**	**I (%) ^a^**	**Range ^c^ (μg/kg)**	**Mean (μg/kg)**						
**OTA**	8(40)	up to 0.8	0.17	8 (40)	up to 7.2	1.08	8 (40)	up to 1.73	0.42				HPLC–FLD	0.02	[45]
**ZEN**	na	–	na	3 (15)	up to 17	14	na	–	na					10	
**FB1**	na	–	na	10 (50)	up to 5960	1930	na	–	na					60	
	**Maize based products (*n* = 17)**			**Rice based products (*n* = 9)**			**Wheat based products (*n* =7)**						**Analytical Method**	**LOQ (µg/kg)**	**Ref.**
	**I (%) ^a^**	**Range ^c^ (μg/kg)**	**Mean ^b^ (μg/kg)**	**I (%) ^a^**	**Range ^c^ (μg/kg)**	**Mean ^b^ (μg/kg)**	**I (%) ^a^**	**Range ^c^ (μg/kg)**	**Mean (μg/kg)**						
**ENNA**	nd	–	nd	nd	–	nd	nd	–	nd				LC–DAD	6	[8]
**ENNA1**	na	423,600	113,000	na	61,400	55,100	1 (14)	up to 46,900	46,900					4	
**ENNB**	nd	–	nd	1 (11)	1050	1050	nd	–	nd					4	
**ENNB1**	1 (5.8)	20,100	20100	1 (11)	600	600	na	up to 79500	79500					5	
**BEA**	nd	–	nd	nd	–	nd	nd	–	nd					5	
**FUS**	nd	–	nd	1 (11)	3900	3900	nd	–	nd					6	

^a^ Incidence of samples ≥LOQ (% of samples ≥LOQ); ^b^ minimum value – maximum; ^c^ Mean value for samples ≥LOQ; *n*: Number of samples; nd: Not detected; na: Not available; DAD: diode array detector; 3-acetyl-deoxynivalenol (3-AcDON), 15-acetyl-deoxynivalenol (15-AcDON), neosolaniol (NEO), diacetoxyscirpenol (DAS), α-zearalenol (α-ZEN), β-zearalanol (β-ZEN), fusaproliferin (FUS), zearalenone-14-glucoside (ZEN-14G), fumonisin B3 (FB3), deoxynivalenol-3-glucoside (DON-3G), enniatin B4 (ENNB4), Nivalenol (NIV).

**Table 4:** Results of the probable daily intake (PDI) assessment of the studied mycotoxins.

Analytes	TDI (µg/kg bw/day)	Barley (*n* = 30)		Maize (*n* = 30)		Rice (*n* = 30)		Wheat (*n* = 3)	
		PDI (µg/kg bw/day)	% TDI	PDI (µg/kg bw/day)	% TDI	PDI (µg/kg bw/day)	% TDI	PDI (µg/kg bw/day)	%
									TDI
**FB1**		0		10.86		0		0	
**FB2**		0		2.05		0		0	
***Sum_FBs_***	2	0	0	12.91	645.4	0	0	0	0
**HT-2**		0		0		0		0.15	
**T-2**		0		0.02		0		0.18	
***Sum_HT2-T2_***	0.1	0	0	0.02	18.26	0	0	0.33	333.8
**DON**	1	0	0	0.46	46.35	0	0	4.92	491.9
**ZEN**	0.25	0	0	0.08	31.97	1.3·10^−3^	0.53	0.85	341.4
**F-X**		0.11		0.21		0		1.27	
**CIT**		0.02		0.02		0		0.14	
**BEA**		0		2.8·10^−3^		0		1.30	
**ENNA**		0		0		0		0.24	
**ENNA1**		0		0.04		0		0.90	
**ENNB**		0		0		0		13.96	
**ENNB1**		0		0.04		0		3.92	

**Table 5:** Sampling information.

Sample Type	Selected Areas	No. of Markets	No. of Samples	Origin
**Barley**	Aint Temouchent	5	10	
	Oran	7	10	**OAIC**
	Tiaret	8	10	
			**Total** = 30	
**Maize**	Aint Temouchent	7	10	
	Oran	6	10	**Imported**
	Tiaret	8	10	
			**Total** = 30	
**Rice**	Aint Temouchent	10	10	
	Oran	7	10	**Imported**
	Tiaret	10	10	
			**Total** = 30	
**Wheat**	Aint Temouchent	6	10	
	Oran	9	10	**NG**
	Tiaret	8	10	
			**Total** = 30	

OAIC: Office Algérien interprofessionnel des céréales, NG: not given.

## Supplementary Materials

The following are available online at https://www.mdpi.com/2072-6651/12/3/194/s1, Figure S1: Matrix effect (%) for each studied mycotoxin extracted from barley, maize, rice and wheat samples (concentration levels: OTA and STE: 25 μg/kg; CIT: 100 μg/kg; FB1, FB2, T-2, HT-2 and ZEN: 250 μg/kg; ENNB, ENNB1, ENA, ENA1 and BEA: 400 μg/kg, DON: 1000 μg/kg and FUS-X: 2500 μg/kg); Figure S2: UHPLC-MS/MS MRM chromatogram of two samples of positive wheat contaminated with (A) DON (1362 µg/kg) and (B) ENNB1 (419 µg/kg); Table S1: Performance characteristics of the UHPLC-MS/MS method for each mycotoxin in barley, maize, rice and wheat samples; Table S2: Recovery (%R), intra-day precision (%RSD_r_) and inter-day precision (%RSD_R_) for barley, maize, rice and wheat samples (*n* = 9); Table S3: Co-occurrence of analysed mycotoxins in wheat and maize samples; Table S4: Monitored ions of the target analytes and MS/MS parameters.
