# Supplementary material for: Multi-Mycotoxin Occurrence and Exposure Assessment Approach in Foodstuffs from Algeria
Source: Toxins (Basel). 2020 Mar 19;12(3):194. doi: 10.3390/toxins12030194 (PMC7150886; doi:10.3390/toxins12030194)
Supplement: Supplementary file 1 [file toxins-12-00194-s001.pdf]

Supplementary Materials: Multi-Mycotoxin Occurrence and Exposure Assessment Approach in Foodstuffs from Algeria

Choukri Khelifa Mahdjoubi, Natalia Arroyo-Manzanares, Nisserine Hamini-Kadar, Ana M. García-Campaña, Kihel Mebrouk and Laura Gámiz-Gracia

Table S1. Performance characteristics of the ultra-high performance liquid chromatography coupled to tandem mass spectrometry (UHPLC-MS/MS) method for each mycotoxin in barley, maize, rice and wheat samples.

Mycotoxin	Barley				Maize				Rice				Wheat			
	Linear Range (ug/kg)	R^2	LOD (ug/kg)	LOQ (ug/kg)	Linear Range (ug/kg)	R^2	LOD (ug/kg)	LOQ (ug/kg)	Linear Range (ug/kg)	R^2	LOD (ug/kg)	LOQ (ug/kg)	Linear Range (ug/kg)	R^2	LOD (ug/kg)	LOQ (ug/kg)
**OTA**	4.0–50	0.991	1.2	4.0	2.6–50	0.988	0.8	2.6	3.4–50	0.992	1.0	3.4	4.8–50	0.991	1.4	4.8
**FB1**	7.5–500	0.986	2.2	7.5	10–500	0.994	3.1	10	9.5–500	0.991	2.8	9.5	2.2–500	0.994	0.7	2.2
**FB2**	9.9–500	0.990	3.0	9.9	2.8–500	0.995	0.9	2.8	8.8–500	0.997	2.6	8.8	9.5–500	0.995	2.9	9.5
**T-2**	2.3–500	0.997	0.7	2.3	24–500	0.991	7.1	24	4.0–500	0.997	1.2	4.0	8.7–500	0.994	2.6	8.7
**HT-2**	4.2–500	0.997	1.3	4.2	4.8–500	0.999	1.4	4.8	4.5–500	0.997	1.4	4.5	4.8–500	0.997	1.5	4.8
**CIT**	9.2–250	0.995	2.8	9.2	8.5–250	0.994	2.5	8.5	4.3–250	0.996	1.3	4.3	9.7–250	0.995	2.9	9.7
**F-X**	132–5000	0.990	40	132	174–5000	0.994	52	174	90–5000	0.993	27	90	138–5000	0.982	41	138
**DON**	92–2000	0.992	28	92	20–2000	0.991	6.0	20	24–2000	0.988	7.1	24	23–2000	0.996	7.0	23
**ZEN**	23–500	0.995	7.0	23	9.8–50	0.989	2.9	9.8	8.4–50	0.996	2.5	8.4	9.3–50	0.991	2.8	9.3
**ENNB**	0.9–500	0.994	0.3	0.9	0.9–500	0.996	0.3	0.9	1.3–500	0.997	0.4	1.3	0.6–500	0.996	0.2	0.6
**ENNB1**	0.6–500	0.992	0.2	0.6	1.5–500	0.991	0.5	1.5	1.4–500	0.993	0.4	1.4	1.6- 500	0.995	0.5	1.6
**BEA**	0.5–500	0.997	0.2	0.5	0.8–500	0.992	0.2	0.8	0.8–500	0.997	0.2	0.8	1.2–500	0.998	0.4	1.2
**ENNA**	1.4–500	0.993	0.4	1.4	1.6–500	0.997	0.5	1.6	1.5–500	0.994	0.4	1.5	2.7–500	0.994	0.8	2.7
**ENNA1**	1.8–500	0.994	0.5	1.8	1.2–500	0.990	0.4	1.2	1.7–500	0.997	0.5	1.7	3.8–500	0.996	1.1	3.8
**STE**	2.9–50	0.980	0.7	2.9	4.4–50	0.992	1.3	4.4	2.6–50	0.992	0.8	2.6	4.4–50	0.996	1.3	4.4

R^2 = determination coefficient; LOD: limit of detection; LOQ : limit of quantification; OTA: ochratoxin A; FB1: fumonisin B1; FB2: fumonisin B2; T-2: T-2 toxin; HT-2: HT-2 toxin; CIT: citrinin; F-X: fusarenon X; DON: deoxynivalenol; ZEN: zearalenone; ENNB: enniatin B; ENNB1: enniatin B1; BEA: beauvericine; ENNA: enniatin A; ENNA1: enniatin A1; STE: sterigmatocystin.

Table S2. Recovery (%R), intra-day precision (%RSD_r) and inter-day precision (%RSD_R) for barley, maize, rice and wheat samples (n=9).

**Mycotoxin.**	**Barley**			**Maize**			**Rice**			**Wheat**		
	**%R**	**%RSD_r**	**%RSD_R**	**%R**	**%RSD_r**	**%RSD_R**	**%R**	**%RSD_r**	**%RSD_R**	**%R**	**%RSD_r**	**%RSD_R**
**OTA**	84.0	6.4	22	78.4	9.1	13	76.6	11	7.3	77.4	7.5	19
**FB1**	100.1	6.9	9.3	103.6	8.7	10	99.1	11	10	95.7	8.0	12
**FB2**	94.8	8.3	7.7	90.0	6.1	14	105.5	9.3	4.3	98.5	5.9	9.9
**T-2**	94.3	4.3	11	87.6	6.5	15	88.9	3.1	4.1	83.4	6.2	12
**HT-2**	87.5	4.2	12	76.5	14	8.6	82.0	5.3	3.3	96.8	5.6	16
**CIT**	42.1	7.7	17	60.0	9.6	20	61.2	4.9	4.8	55.1	11	23
**F-X**	84.4	5.5	7.8	81.1	8.3	18	82.4	7.3	8.5	87.6	6.5	22
**DON**	83.6	6.0	19	84.5	5.0	7.9	80.5	9.1	4.4	83.8	5.5	14
**ZEA**	98.9	7.6	20	89.5	8.3	11	94.8	5.5	7.9	87.1	6.9	9.9
**ENNB**	91.9	2.7	6.1	88.3	3.9	5.7	94.1	3.0	1.7	88.6	2.4	6.7
**ENNB1**	94.4	2.5	9.8	90.3	5.1	8.4	95.3	2.8	2.0	89.7	2.2	6.3
**BEA**	84.4	1.9	6.2	88.0	5.3	3.4	91.7	3.8	2.3	91.9	5.3	5.7
**ENNA1**	98.6	3.1	6.0	93.4	3.4	9.5	105.0	4.1	4.9	93.7	2.9	7.9
**ENNA**	98.0	1.9	6.9	90.4	4.6	15	105.3	2.7	3.4	93.9	3.2	5.6
**STE**	76.5	7.3	16	70.6	12	14	65.5	11	17	70.0	8.7	8.4
**AVERAGE**	**87.5**	**8.6**	**11.8**	**84.8**	**7.3**	**11.4**	**88.5**	**6.2**	**5.7**	**86.2**	**5.8**	**11.9**

Levels: OTA and STE: 25 μg/kg; CIT: 100 μg/kg; FB1, FB2, T-2, HT-2 and ZEA: 250 μg/kg; ENNB, ENNB1, ENNA, ENNA1 and BEA: 400 μg/kg, DON: 1000 μg/kg and F-X: 2500 μg/kg.

Table S3. Co-occurrence of analysed mycotoxins in wheat and maize samples.

**Sample Code**	**Mycotoxins Concentration (µg/kg)**											**Number of Mycotoxins Found**
	**DON**	**HT-2**	**T-2**	**ZEN**	**F-X**	**CIT**	**BEA**	**ENNA**	**ENNA1**	**ENNB**	**ENNB1**	
**Wheat 10**	114.2	–	21.34	–	–	–	–	–	–	–	–	**2**
**Wheat 8**	68.3	–	20.74	–	–	–	–	–	–	1.16	–	**3**
**Wheat 9**	92.1	–	34.65	–	–	–	–	–	–	330	282	
**Wheat 5**	1072	–	33.80	–	–	–	4.30	43.7	–	–	–	**4**
**Wheat 25**	815	–	17.54	–	–	–	18.5	8.87	–	–	–	
**Wheat 2**	–	–	25.01	–	156	13.8	–	–	–	85.6	67.1	
**Wheat 3**	–	–	29.96	10.7	–	22.0	–	–	–	31.6	19.5	
**Wheat 7**	–	–	30.38	12.7	–	9.75	–	–	–	65.6	49.5	
**Wheat 22**	396	–	17.48	–	–	32.3	2.80	–	75.9	–	–	**5**
**Wheat 23**	226	–	16.89	–	–	–	297	–	395	–	265	
**Wheat 26**	930	–	16.94	15.0	–	–	19.4	–	102	–	–	
**Wheat 29**	280	–	16.88	–	–	12.2	17.3	–	100	–	–	
**Wheat 1**	735	–	47.25	22.8	–	–	–	–	22.9	2779	148	
**Wheat 6**	145	–	27.16	13.1	–	–	–	–	7.04	44.7	31.1	
**Wheat 21**	1363	36.7	20.74	20.3	–	–	10.7	–	170	–	–	
**Wheat 4**	270	–	30.55	–	–	13.2	–	–	3.98	2337	197	**6**
**Wheat 24**	263	–	16.88	9.57	–	–	443	–	28.3	–	299	
**Wheat 27**	650	27.0	19.93	–	–	16.0	8.68	–	65.0	–	–	
**Wheat 11**	224	–	17.06	72.6	–	30.4	127	–	–	1828	91.0	
**Wheat 16**	97.3	–	19.87	40.1	–	11.8	27.8	–	–	159	132	**7**
**Wheat 20**	770	–	17.00	212	–	–	348	9.45	–	2926	249	
**Wheat 12**	578	9.82	17.48	163	–	–	473	17.9	–	3865	332	
**Wheat 14**	724	–	16.64	201	–	10.6	263	8.36	–	2809	151	
**Wheat 17**	945	–	17.18	257	–	14.7	84.8	87.6	–	5288	4569	
**Wheat 18**	932	–	17.06	254	–	14.0	486	22.4	–	2540	215	
**Wheat 28**	285	–	17.42	12.2	139	30.4	33.2	–	160	–	65.3	**8**
**Wheat 13**	87.0	14.0	18.81	37.5	–	–	133	–	197	50.3	36.0	
**Wheat 19**	1090	8.35	17.21	295	159	10.1	280	–	–	–	168	
**Wheat 30**	1189	12.6	16.82	11.2	–	–	28.6	–	155	2006	31.8	
**Wheat 15**	1053	18.9	17.54	285	–	10.6	57.2	–	18.40	2877	2448	**9**

Table S3. *cont.*

Sample Code	Mycotoxin Concentration (µg/kg)										Number of Mycotoxins Found
	DON	FB1	FB2	T-2	ZEN	F-X	CIT	BEA	ENNA1	ENNB1	
**Maize 9**	–	–	–	25.0	–	303	–	–	–	–	**2**
**Maize 10**	–	289	–	25.4	–	267	–	–	–	–	**3**
**Maize 29**	–	16,082	240	25.0	–	–	13.1	5.63	–	–	**5**
**Maize 16**	–	1263	7319	25.0	–	314	–	0.88	–	–	
**Maize 8**	–	9364	84.7	24.5	–	345	–	1.84	–	–	
**Maize 17**	–	1420	181	25.2	–	398	10.1	–	–	–	
**Maize 21**	–	5053	237	24.9	–	–	10.6	2.75	–	–	
**Maize 24**	422	12,347	–	25.2	–	253	–	4.31	–	–	
**Maize 30**	–	3389	220	25.0	–	–	9.07	1.93	–	–	
**Maize 6**	–	42,143	6735	25.1	–	–	9.97	1.49	–	–	
**Maize 26**	–	656	27.5	25.2	–	–	9.40	4.34	–	–	
**Maize 3**	–	31,122	4711	24.5	–	259	12.2	1.52	–	–	**6**
**Maize 7**	–	1466	822	24.7	–	315	8.55	2.13	–	–	
**Maize 18**	–	5893	113	24.8	–	236	11.8	0.91	–	–	
**Maize 19**	–	930	122	24.6	–	368	15.0	0.85	–	–	
**Maize 22**	–	3998	1600	24.6	–	477	10.4	2.74	–	–	
**Maize 28**	368	28,930	5167	24.8	–	–	273	7.90	–	–	
**Maize 1**	–	4186	184	25.7	–	198	8.79	31.4	–	107	**7**
**Maize 5**	–	39,038	8603	25.0	–	352	10.8	1.03	11.5	–	
**Maize 14**	820	2130	353	24.6	–	255	255	0.96	–	–	
**Maize 23**	47.6	10,967	1991	25.0	–	228	12.4	6.06	–	–	
**Maize 25**	261	2942	148	24.6	–	391	10.8	1.67	–	–	
**Maize 15**	913	41,027	3505	25.0	20.4	248	273	–	–	–	
**Maize 20**	866	17,827	403	24.8	579	211	10.8	–	–	–	
**Maize 2**	887	17,990	3060	25.4	–	265	14.0	11.9	–	15.0	**8**
**Maize 12**	2055	27,326	6000	25.0	22.9	194	16.0	0.94	–	–	
**Maize 13**	482	33,111	7726	25.1	44.7	177	30.0	0.92	–	–	
**Maize 27**	103	34,816	6118	25.0	13.3	236	12.1	5.09	–	–	
**Maize 4**	536	18,254	2964	25.3	40.4	281	14.5	1.14	53.9	–	**9**
**Maize 11**	456	30,411	6680	24.9	39.7	270	12.7	0.95	103	–	

Table S4. Monitored ions of the target analytes and MS/MS parameters.

Compound	Elemental composition	Retention time (min)	Precursor ion (m/z)	Molecular ion	DP [a]	EP [a]	CEP [a]	Product Ions [b]	CE [a]	CXP [a]
DON	$C_{15}H_{20}O_6$	1.27	297.1	$[M+H]^+$	36.0	5.5	16.0	249.2(Q)	17.0	4.0
								161.0(I)	29.0	4.0
F-X	$C_{17}H_{22}O_8$	1.46	355.1	$[M+H]^+$	26.0	12.0	18.0	147.7(Q)	23.0	4.0
								137.1(I)	31.0	4.0
CIT	$C_{13}H_{14}O_5$	2.28	251.2	$[M+H]^+$	26.0	11.0	18.0	233.0(Q)	23.0	23.0
								204.8(I)	73.0	10.0
HT-2	$C_{22}H_{32}O_8$	2.74	442.0	$[M+NH_4]^+$	21.0	5.5	21.0	262.8(Q)	22.0	8.0
								215.4(I)	19.0	4.0
FB 1	$C_{34}H_{59}NO_{15}$	3.00	722.2	$[M+H]^+$	71.0	10.0	30.0	334.2(Q)	51.0	6.0
								352.2(I)	47.0	6.0
T-2	$C_{24}H_{34}O_9$	3.17	484.0	$[M+NH_4]^+$	21.0	10.0	22.0	215.0(Q)	22.0	4.0
								185.0(I)	29.0	4.0
ZEN	$C_{18}H_{22}O_5$	3.53	319.0	$[M+H]^+$	26.0	8.0	20.0	282.9(Q)	19.0	4.0
								301.0(I)	15.0	10.0
OTA	$C_{20}H_{18}ClNO_6$	3.62	404.0	$[M+H]^+$	41.0	7.5	16.0	238.9(Q)	31.0	6.0
								102.1(I)	91.0	6.0
STE	$C_{18}H_{12}O_6$	3.68	325.1	$[M+H]^+$	66.0	3.5	26.0	281.0(Q)	43.0	4.0
								310.0(I)	37.0	4.0
FB 2	$C_{34}H_{59}NO_{14}$	3.81	706.2	$[M+H]^+$	71.0	10.5	20.0	336.3(Q)	43.0	14.0
								318.3(I)	45.0	12.0
ENNB1	$C_{33}H_{57}N_3O_9$	5.20	640.4	$[M+H]^+$	81	8.50	18.0	196.2(Q)	35.0	4.0
								214.2(I)	37.0	4.0
ENNB	$C_{34}H_{59}N_3O_9$	5.30	654.4	$[M+H]^+$	81	7.50	30.0	196.4(Q)	39.0	6.0
								210.1(I)	33.0	6.0
BEA	$C_{45}H_{57}N_3O_9$	5.40	784.5	$[M+H]^+$	81	9.00	26.0	244.2(Q)	39.0	4.0
								262.2(I)	37.0	4.0
ENA 1	$C_{35}H_{61}N_3O_9$	5.50	668.4	$[M+H]^+$	81	10.5	18.0	210.2(Q)	35.0	6.0
								228.2(I)	35.0	4.0
ENA	$C_{36}H_{63}N_3O_9$	5.60	682.4	$[M+H]^+$	76	9.00	26.0	210.2(Q)	35.0	4.0
								228.2(I)	35.0	4.0

[a] Declustering potential (DP), entrance potential (EP), collision cell entrance potential (CEP), collision cell exit potential (CXP) and collision energy (CE). All expressed in voltage. [b] Product ions: (Q) transition used for quantification, (I) transition employed to confirm the identification.

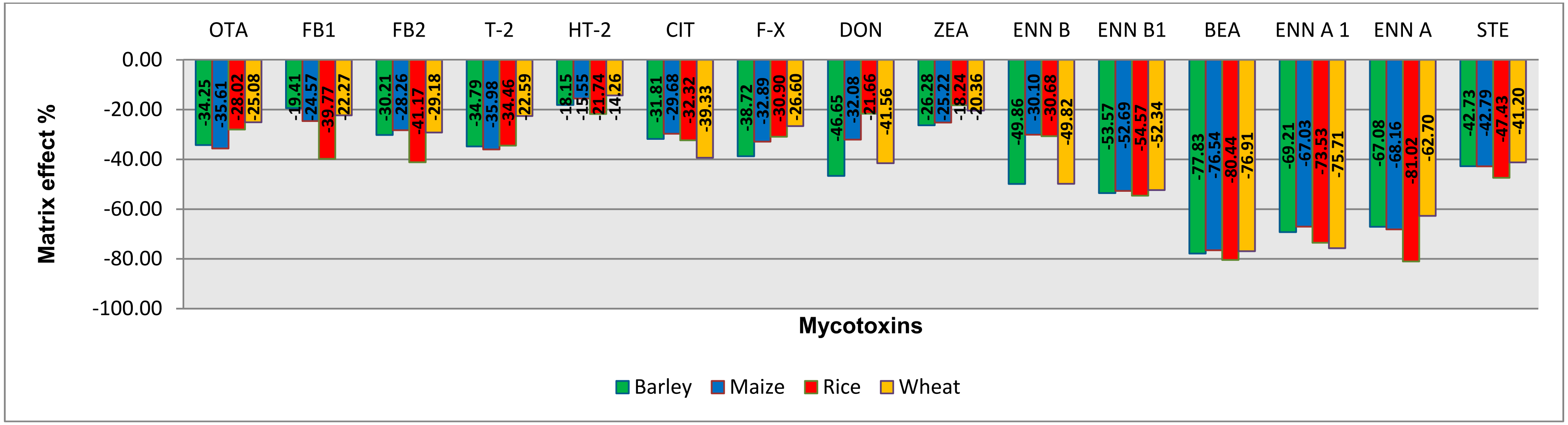

Figure S1. Matrix effect (%) for each studied mycotoxin extracted from barley, maize, rice and wheat samples (concentration levels: OTA and STE: 25 µg/kg; CIT: 100 µg/kg; FB1, FB2, T-2, HT-2 and ZEN: 250 µg/kg; ENNB, ENNB1, ENNA, ENNA1 and BEA: 400 µg/kg, DON: 1000 µg/kg and F-X: 2500 µg/kg).

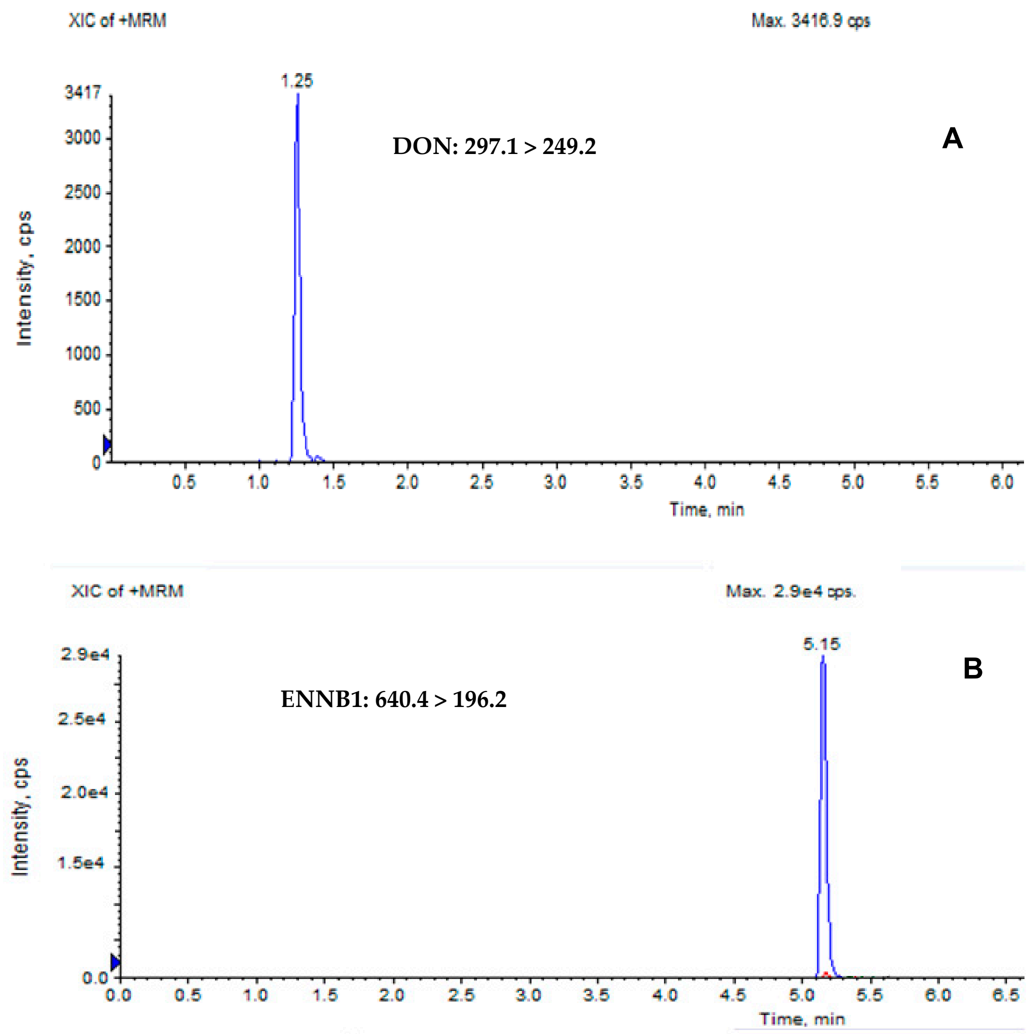

Figure S2. UHPLC-MS/MS multiple reaction monitoring (MRM) chromatogram of two samples of positive wheat contaminated with (**A**) DON (1362 µg/kg) and (**B**) ENNB1 (419 µg/kg).
